# How does CEO power and overconfidence affect the systemic risk of China’s financial institutions?

**DOI:** 10.3389/fpsyg.2022.847988

**Published:** 2022-09-23

**Authors:** Yingying Chen, Adnan Safi, Yasir Zeb

**Affiliations:** ^1^School of Economics, Qingdao University, Qingdao, Shandong, China; ^2^Department of Management Sciences, Virtual University of Pakistan, Lahore, Pakistan; ^3^School of Business, Qingdao University, Qingdao, Shandong, China

**Keywords:** systemic risk, ∆CoVaR, CEO power, overconfidence, financial institutions, state-owned enterprises, China

## Abstract

The purpose of this paper is two-fold. First, this study measures the contribution of banks and non-bank financial institutions toward the systemic risk of China. Second, the present study investigates the relationship between CEO power, CEO overconfidence, and systemic risk. This study uses the Delta Conditional Value-at-Risk (∆CoVaR) method to measure the systemic risk contribution of firms listed on the Shenzhen and Shanghai stock exchanges over a period of 2006–2018. The results show that non-bank financial institutions are systemically more important compared to banks. We employed fixed-effect regression analysis to show that banks with overconfident CEOs increase the firm’s systemic risk. The results also confirm that powerful CEOs enhance the contribution of non-bank financial institutions to systemic risk, whereas CEO power’s impact was significant only for non-state-owned banks. The findings were further validated by the robustness test results obtained using the two-stage least squares approach. These findings are important for constructing regulations to reduce the contribution of firms to systemic risk.

## Introduction

China’s economy has shown significant growth during the past two decades and has become a major economic force in the world, exhibiting great potential (Xiong et al., 2020). However, there have been considerable fluctuations during the last 5 years, and the economic growth of China has fallen from 9.9 in 1995–2010 to 7.0 in 2016–2019 (World Bank, 2020). Financial firms play a key part in a country’s economic growth. Despite China’s rapid economic growth, financial institutions have experienced many lows and highs (Safi et al., 2021). The financial crisis of 2007–2008 brought forth intensive debate about the financial sector’s systemic nature. The global financial crisis exposed the system’s fragility, where a decline of one industry impacted other industries and consequently hindered economic growth (Acharya et al., 2017). Furthermore, the crises spread to other countries and decelerated their economic growth. Therefore, it draws attention from governments, monitoring agencies, and researchers to identify the key factors contributing to systemic risk. Systemic risk is the collapse of the whole system and can be explained as the risk that exposes the whole system to severe system losses (Safi et al., 2021). After the 2007–2008 financial crisis and the 2015–2016 China stock market collapse, researchers, regulators, and policymakers have focused their attention on systemic risk to measure it accurately and reduce the influence of systemic risk.

Many studies have examined the aspects that affect systemic risk, and studies have employed different measures to estimate systemic risk, such as systemic risk index (SRISK), Marginal Expected Shortfall (MES), and delta Conditional Value at Risk ( ΔCoVaR ; Kleinow et al., 2017). Numerous studies have explored the factors that affect financial institutions’ systemic risk with the primary objective of decreasing systemic risk and improving the financial system’s stability. The firm-level key determinants of systemic risk have been thoroughly studied for the financial system (e.g., Ilin and Varga, 2015; Zeb and Rashid, 2019; Braiek et al., 2020; Cao, 2020; Choi et al., 2020; Kapinos, 2020; Wen et al., 2020). However, studies have ignored the behavioral perspective, which can provide useful new insights, including the overconfidence and CEO power perspective. Therefore, this research’s primary objective is to analyze CEO overconfidence and CEO power’s influence on the firm’s systemic risk in the context of China’s financial sector.

In behavioral finance, overconfident executives have a considerable influence on decision-making in a firm. Overconfidence can be described as managers’ tendency to overestimate their capability and their relative probability of success. For example, overconfident executives tend to underestimate the volatility of unexpected occurrences or overestimate the potential returns on their assets. Heaton (2005) designed a simplified model to illustrate the volatility of investment cash flow due to over-confident executives. Malmendier and Tate (2005) conducted a study using executive stock options to assess CEO overconfidence and noticed that CEOs are often more inclined toward internal cash flow for over-confident investment. According to Malmendier and Tate (2008), firms with overconfident CEOs also prefer to take on mergers that destroy value as they overestimate their capability to generate returns.

The present study also focuses on CEO power as a determining factor of systemic risk, as earlier research studies have ignored the effect of powerful CEOs on risk. However, studies have revealed the effect of CEO power on firm conduct and efficiency, including the firm’s performance (Adams et al., 2005). Financial institutions are vulnerable to risk-taking because of their high leverage, the restricted market discipline of creditors, and the potential to quickly and opaquely raise their assets’ riskiness. Also, financial institutions’ failure can be devastating for investors and can have a detrimental impact on the economy. Therefore, it is not surprising that a substantial regulatory and academic discussion on the degree to which governance deficiencies play a role in financial institutions’ systemic risk contributions. Studies have shown that the financial institution’s instability during the recession that began in 2008 was triggered by a rise in unnecessary risk (Deyoung et al., 2013).

This study measures the financial institutions’ systemic risk contribution by categorizing them into banks and non-bank financial institutions. Furthermore, this study adds to the existing literature in many ways; first, this study explores the linkage between CEO overconfidence and systemic risk for financial institutions listed in Shenzhen and Shanghai stock exchanges over a period of 2006–2018. Second, the present study also inspects the influence of CEO power on systemic risk, as limited studies have investigated the linkage between powerful CEOs and Systemic risk for China. Third, the present study has divided banks and non-bank financial firms into state-owned and non-state-owned enterprises to have a detailed overview of their systemic risk contribution and provide an in-depth empirical analysis of the linkage between CEO Power, overconfidence, and systemic risk. Detailed analyses may help develop regulations to reduce the systemic risk contribution of financial firms.

The rest of the article is structured as follows: the next part gives the literature survey and hypothesis development, the third part of the study explains the methodology, variable, and data description, the fourth part gives the empirical results, and the last is the conclusion and recommendations.

## Literature review and hypothesis development

A number of studies have been conducted to examine the factors that affect risk in the financial sector to avoid extreme moments before it occurs (Tripe et al., 2009; McKelvey and Andriani, 2010; Kanno, 2018; Schnatterly et al., 2018; Wen et al., 2020; Matenda et al., 2021). For example, studies have explored the role of governance (Chavarín, 2020), bank risk shifting and diversification (Alaabed et al., 2016; Batten and Vo, 2016), information system security (Koskosas, 2008; Jakšič and Marinč, 2018), operational risk (Blacker, 2000), contagion risk (Der Su, 2018), managerial practices (Al Khattab et al., 2008), and organizational culture (Imran et al., 2021) in the financial sector to mitigate the risk. However, studies mainly focus on firm-level factors and have ignored the CEOs’ impact on systemic risk.

### CEO overconfidence and systemic risk

Financial Institutions are distinctive due to their systemic and interrelated nature. Any volatility, insolvency, or disruption in an individual institution will affect others in the financial sector (Drehmann and Tarashev, 2013). Several research studies have investigated the influence of different firm-level factors on systemic risk, and studies have shown firm’s size, loan ratio, leverage ratio, and other firm-level factors as key determinants of systemic risk (Ilin and Varga, 2015; Bougheas and Kirman, 2018; Peltonen et al., 2019). However, a few studies have considered behavioral biases, and limited studies have explored the influence of CEO behavioral biases on a firm’s systemic risk. It is difficult to refute the role of executives of financial firms and their confidence in potential policy outcomes. Ho et al. (2016) assert that over-confident CEOs overestimate their loan projections, put less weight on potential losses, and, as a result, ease lending criteria. Moreover, firms with overconfident CEOs overestimate their ability and likelihood of loan recovery and therefore allow a low loan loss provision (Black and Gallemore, 2013). Overconfidence also drives managers’ preference for debt maturity when it comes to financing decisions (Huang et al., 2016). A study conducted by Landier and Thesmar (2008) indicates that short-term debt is more likely to be taken on by overconfident CEOs. Similarly, the study of Huang et al. (2016) revealed empirically that firms with overconfident CEOs assume that an increase in short-term debt will increase stakeholders’ value as they overestimate the prospect that short-term debt can be refinanced with reduced costs and future positive news.

Niu (2010) conducted a research study that showed the linkage between overconfident CEOs and a firm’s risk-taking by examining the financial institutions and found a higher standard deviation in stock returns in those financial institutions that are led by overconfident CEOs. Ma (2014) investigated how financial institutions’ pre-crisis investments corresponded to the overconfidence of their CEOs and confirmed that institutions with overconfident CEOs tend to have more real estate loans. A study by Ho et al. (2016) used the stock-based option as a measure for CEO overconfidence to analyze how, during crises, managerial overconfidence explains the considerable variability in financial institutions’ risk-taking behavior. They found that in the run-up to a crisis, financial institutions with over-confident CEOs are more likely to reduce loan standards and raise the firm’s leverage, making them more susceptible to the impact of the crisis.

Safi et al. (2021) conducted a research study on CEO overconfidence and systemic risk using Chinese firms’ data. The result of their study showed that banks that have overconfident CEOs have a higher systemic risk. Similarly, Lee et al. (2020) showed in a study on banks that CEOs’ overconfidence is positively associated with systemic risk. Moreover, this influence is significant during a period of crisis. A similar study conducted by Liu et al. (2020) on US banks revealed that compared to non-overconfident CEOs, the systemic risk of firms with overconfident CEOs is higher. Their study further argues that overconfident CEOs can negatively impact other firms in the sector. Overconfident CEOs raise their financial institution’s risk with investment and financing decisions. Moreover, overconfident CEOs overestimate the firm’s growth and performance to refinance debt with lower rates and expose the firm to unwanted risk. Based on the above discussion and existing literature, it can be said that CEO overconfidence contributes to systemic risk. Hence, the hypothesis is given as follows:

*Hypothesis 1*: Financial institutions with Overconfident CEOs have a higher systemic risk contribution.

### CEO power and systemic risk

There is very little literature on the behavioral characteristics and impact of a CEO, particularly a CEO power on performance and financial institutions’ risk-taking. The theory of managerial power and organizational theory provides contradictory perspectives concerning the association between CEO power and firm risk. On the one hand, the Agency’s theory indicates that CEOs are justified in choosing safer assets than the ones preferred by shareholders, as CEO assets include human, tangible, and financial capital invested in the institutions they run, while risk can be diversified by shareholders in the financial market (Pathan, 2009). On the other side, as per organizational theory, the power delegated to a CEO, which indicates a unity of command and guarantees the highest performance in decision-making and execution, helps bring into line the executive’s interest with that of stakeholders.

The empirical results are mixed about the effect of CEO Power on financial institutions’ systemic risk. Pathan (2009) argues that board decisions are influenced by powerful CEOs in US financial institutions so that risk-taking is reduced. A study conducted by Victoravich et al. (2011) showed that CEO power mitigate risk-taking in the United States financial institutions while managing the equity compensation of CEO. Additionally, their study argues that CEOs impact the decision-making of the boards in reducing risk. In contrast, Lewellyn and Muller-Kahle's (2012) study showed an association between powerful CEOs and overly risky lending practices in many US firms, out of which half of them were engaged in subprime lending. While institutions with powerful CEOs adopt policies that lead to more risky results and guide board decisions to adopt risky policies (Adams et al., 2005). Altunbaş et al. (2020) found that the CEO power is linked with a rise in risk-taking in the financial sector, and there is little proof that financial institutions’ board characteristics mitigate this risk associated with power. They also find evidence that powerful CEOs are more likely to invest in a high-risk project when they have an extensive network and long tenure. CEOs could take advantage of the information they have by using their wider networks, growing information asymmetries within the organization, and alleviating the adverse selection issue that is among the reasons for excessive risk-taking. Likewise, Shahab et al. (2020) study also concluded that firms with powerful CEOs have a high probability of stock price crash as CEOs tend to hide bad news from the investors to save their career and when the bad news piles up and can no longer be contained they are released at once causing a sharp decline in stock prices. In light of the above debate, the hypothesis is as follows:

*Hypothesis 2*: Powerful CEOs have a positive impact on the firm’s systemic risk contribution.

## Methodology

### Data description

The present study measures banks and non-bank financial institutions’ contribution to the systemic risk of China. Furthermore, this study also examines CEO overconfidence and CEO power as a cause of systemic risk. For this purpose, data is collected from CSMAR and WIND financial terminal databases comprising of banks and non-bank financial institutions (NBFIs) listed in the Shenzhen and Shanghai stock exchanges over a period of 2006–2018. The data includes 28 banks and 59 NBFIs. In this study, the data collected are further divided into state-owned and non-state-owned enterprises, including 8 and 34 state-owned banks and NBFIs; non-state-owned enterprises include 20 banks and 25 NBFIs. The dependent variable in this study is systemic risk measured using Adrian and Brunnermeier's (2016) method of delta conditional value at risk $\left(\Delta CoVaR\right)$ , the main independent variables are CEO overconfidence (OCE) calculated using earning forecast bias proxy and CEO power (PWE) measured using an index based on CEO tenure and duality. The control variables selected in this research study are the firm’s size (Size) calculated as a log of total assets, firm’s loan ratio (LNR) calculated using the loan to asset ratio, leverage ratio (Levr) calculated as debt to equity ratio, and return on total assets (ROA) calculated as net income to firm’s total assets. In this study, we employed the fixed-effect panel regression method to determine the linkage between CEO power, CEO overconfidence, and systemic risk. Based on the research study of Safi et al. (2022), the econometric model is given as:


(1) $$SysRISK_{i,t}=\delta_{1}OCE_{i,t}+\delta_{2}PWE_{i,t}+\delta Control_{i,t}+\varepsilon_{i,t}$$

Where, $SysRISK_{i,t}$ gives the systemic risk measured using ΔCoVaR method proposed by Adrian and Brunnermeier (2016), OCE*_i,t_* is the CEO overconfidence, PWE_i,t_ is the CEO power measured using an index, whereas the $Control_{i,t}$ indicates the control variables (i.e., size, loan ratio, leverage, and ROA).

### Overconfidence measure

Based on the study of Wang et al. (2008); He et al. (2019), and Safi et al. (2021), we employed corporate earnings forecasts bias as an indicator of CEO overconfidence. A firm’s CEO is regarded as overconfident if the actual earnings are below the projected or expected earnings that they forecasted. We created a dummy variable with a value of “1” and “0.” A value of one is given to overconfident CEOs who overestimate the firm’s actual earnings and forecast the earnings more by overestimating their performance, and a value of zero is given if the firms’ actual earnings are equal or greater to the firm’s projected earnings.

### CEO power measure

We have taken two indicators to measure CEO power following previous research studies. The first indicator to measure CEO power is the tenure of the CEO, which signifies the number of years in the same role that the CEO has held, with power seen as growing with the duration of tenure since tenure creates autonomy in decision-making. Furthermore, studies show that longer tenure is related to a decrease in career concerns (Altunbaş et al., 2020), indicating that tenure is strongly correlated with risk-taking. The second measure is duality, whether the CEO holds a senior title or is also the chairman. This indicator has been widely used in the literature, and the CEO is regarded as powerful if he/she is also chairman of the firm (Li et al., 2016; Safi et al., 2021).

### Systemic risk measure

Previous studies have put forward different measures to calculate a firm’s systemic risk contributions, such as Systemic Risk Index (SRISK), Conditional Value-at-Risk (CoVaR), and Marginal Expected Shortfall (MES). Based on the study of Kleinow et al. (2017) and Zeb and Rashid (2019), we employed a market-based approach using stock return data to measure systemic risk by employing Adrian and Brunnermeier's (2016) Delta Conditional Value at Risk ( ΔCoVaR ) method for Chinese banks and non-bank financial institutions (NBFIs). At present, the ΔCoVaR measure is one of the popular methods for tail measurement. CoVaR calculates the financial institution’s value at risk (VaR) when a financial firm is in distress, and the ΔCoVaR shows the firm’s systemic risk contributions and is estimated as the difference in the CoVaR conditional on the firm being in distress and the median state. The VaR for each firm “j” in quantile “q” can be expressed as $aR_{q}^{j}$ , then the VaR for the financial system when a firm is in distress can be expressed as $CoVaR_{q}^{sys|X^{j}=VaR_{q}^{j}}$ , the equation is given as:


(2) $$\mathrm{Pr}\left(X^{sys}\leq CoVaR_{q}^{sys\left|X^{j}=VaR_{q}^{j}\right.}\left|X^{j}=VaR_{q}^{j}\right.\right)=q$$

Then financial firms’ systemic risk contribution can be given as:


(3) $$CoVaR_{q}^{sys|j}=CoVaR_{q}^{sys|X^{j}=VaR_{q}^{j}}-CoVaR_{q}^{sys|X^{j}=Median}$$

Following the study of Adrian and Brunnermeier (2016), using financial market data, the ΔCoVaR model can be given as:


(4) $$X_{t}^{j}=\alpha^{j}+\delta^{j}RC_{t-1}+\varepsilon_{t}^{j}$$

(5) $$X_{t}^{sys}=\alpha^{sys|j}+\gamma^{sys|j}X_{q}^{j}+\delta^{sys|j}RC_{t-1}+\varepsilon_{q,t}^{sys|j}$$

In equations 4, 5, $X_{t}^{j}$ shows the returns of the banks and non-bank financial institutions j at the time t, $X_{t}^{sys}$ shows the rate of return of the whole financial system “sys” at time t, $\gamma^{sys|j}X_{t}^{j}$ shows the distress of banks and non-bank financial firms on the financial system, $\delta^{sys|j}$ calculates the impact of external factors on the system, whereas $RC_{t-1}$ are the country-level risk factors. The risk factors included in this study are credit spread estimated as the change in corporate bond rate AAA and 10 years treasury bond rate, Capital market volatility calculated as the volatility yield rate of bonds, yield curve estimated as the difference in 10 years bond rate and 3 month treasury bond rate, market liquidity estimated as the difference between 3 month interbank offered rate and treasury bond rate, the Chinese implied volatility index and the percent change in 3 month treasury bill rate. The quantile regression can be given as:


(6) $$VaR_{q,t}^{j}={\hat{\alpha }}_{q}^{j}+{\hat{\delta }}_{q}^{j}RC_{t-1}$$

(7) $$CoVaR_{q,t}^{j}={\hat{\alpha }}_{q}^{sys|j}+{\hat{\gamma }}_{q}^{sys|j}VaR_{q,t}^{j}\left(q\right)+{\hat{\delta }}_{q}^{sys|j}RC_{t-1}$$

Hence, $\Delta CoVaR_{q,t}^{sys|j}$ can be given as:

$$\Delta CoVaR_{q,t}^{sys|j}=CoVaR_{t}^{j}-CoVaR_{50,t}^{j}={\hat{\gamma }}_{q}^{sys|j}\left(VaR_{q,t}^{j}\left(q\right)-VaR_{50,t}^{j}\right)$$

In the above equation, $\Delta CoVaR_{q,t}^{sys|j}$ shows the rate at which the firms transfer risk to the financial system.

## Empirical results

In this part, the empirical results obtained *via* various statistical analyses are described. The descriptive statistics of the financial institutions’ contributions to China’s systemic risk are given in Table 1. The descriptive statistics show that NBFIs contribute more to systemic risk with a mean value of −0.321 compared to banks −0.306. Moreover, non-state-owned enterprises’ contribution to systemic risk is higher compared to state-owned firms. For banks, non-state-owned firms contribute more with a mean value of −0.307 compared to state-owned −0.304. Similarly, the non-bank financial institutions, state-owned, contributed less with a mean value of −0.314 than non-state-owned enterprises −0.331.

**Table 1:** Financial institutions systemic risk ( Δ CoVaR).

Institutions	Mean	Std. dev	Min	Max
Financial System	−0.317	0.0569	−0.2082	−0.4989
Banks	−0.306	0.0627	−0.2093	−0.4930
Banks (SOE)	−0.304	0.0607	−0.2093	−0.4638
Banks (Non-SOE)	−0.307	0.0632	−0.2099	−0.4930
Non-bank financial institutions	−0.321	0.0536	−0.208	−0.4989
Non-bank financial institutions (SOE)	−0.315	0.0578	−0.2082	−0.4989
Non-bank financial institutions (Non-SOE)	−0.332	0.0443	−0.2177	−0.4859

SOE represents the state-owned enterprises, and non-SOE are the non-state-owned enterprises.

**Figure 1:**
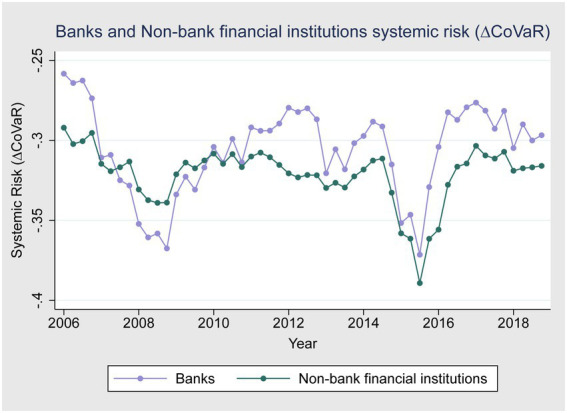
Banks and non-bank financial institutions systemic risk.

**Figure 2:**
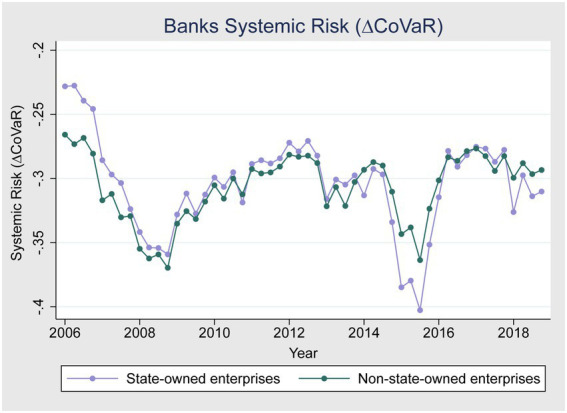
Banks systemic risk (SOE and Non-SOE).

**Figure 3:**
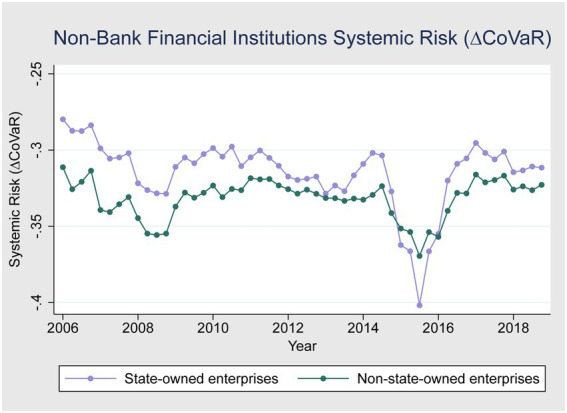
Systemic risk of non-bank financial institutions (SOE and Non-SOE).

Figures 1–3 give a more detailed overview of the contribution of banks and non-bank financial institutions to systemic risk from 2006 to 2018. Figure 1 shows that banks contributed more to systemic risk during the global financial crises of 2007–2008 compared to non-bank financial institutions. Figure 2 shows that the systemic risk of non-state-owned banks is higher than state-owned enterprises during the crises, as state-owned enterprises are highly regulated and financially backed by the Chinese government and do not have performance pressure. Figure 1 also shows that during the 2015–2016 China stock market crises, non-banking financial institutions’ systemic risk contribution was higher compared to banks. One possible reason for this could be that international and domestic regulations were made stricter after the global financial crisis to avoid future crises. Figure 3 shows that state-owned enterprises contributed more to systemic risk during the 2015–2016 stock market turbulence than non-state-owned enterprises.

Table 2 gives the regression results for banks. The results reveal that CEO overconfidence significantly positively influences systemic risk. Models (1) to (3) show results for banks as a whole, state, and non-state-owned enterprises, indicating that CEO overconfidence significantly increases the firm’s systemic risk with coefficient values of 0.158 for overall banks, 0.210 for state-owned enterprises, and 0.238 for non-state-owned enterprises. These results can be explained by the theory of behavioral finance that overconfident CEO overestimates their returns and invest in projects that involve high risk, which leads to an increase in firm risk and ultimately enhances the firm’s systemic risk contribution (Ben-David et al., 2013). Moreover, the results also indicate a significant positive association between the size of the firm and systemic risk indicating that bigger firms have a higher systemic risk, and this effect is higher in non-state-owned banks compared to state-owned. These results are in line with the findings of Lee et al. (2020) and Safi et al. (2021).

**Table 2:** Regression analysis for banks.

	Dependent variable: systemic risk ( ΔCoVaR )		
	(1) Banks	(2) SOE	(3) Non-SOE
OCE_t_	0.158^***^	0.210^***^	0.238^***^
	(0.030)	(0.053)	(0.088)
PWE_t_	0.162	0.072	0.086^**^
	(0.149)	(0.067)	(0.031)
Size_t_	0.142^***^	0.190^***^	0.388^***^
	(0.028)	(0.039)	(0.135)
LEVR_t_	0.002	0.017	−0.014
	(0.009)	(0.013)	(0.037)
ROA_t_	−0.114^***^	−0.132^***^	−0.244^***^
	(0.018)	(0.026)	(0.038)
LNR _t_	0.300^***^	0.295	0.061^*^
	(0.030)	(0.068)	(0.036)
N	810	188	622
adj. R2	0.821	0.806	0.81
Hausman test (value of *p*)	0.001	<0.001	<0.001
F (value of *p*)	<0.001	<0.001	<0.001

The asterisks *, **, and *** indicate the significance levels at 10%, 5%, and 1%, respectively.

Table 2 also gives the results for CEO power and systemic risk. Model (1) to (3) presents the results for banks, state-owned and non-state-owned banks. The findings indicate that CEO power has a positive but insignificant effect on overall banks and state-owned banks, but the association between CEO power (PWE) and systemic risk ( ΔCoVaR ) is positive and significant for non-state-owned banks with a coefficient value of 0.086. This indicates that non-state-owned banks with powerful CEOs have a higher systemic risk contribution as compared to non-powerful CEOs. The logical explanation for this is that Powerful CEOs may adopt policies that lead to high risk and guide board decisions to adopt risky policies (Adams, Almeida & Ferreira,2005). Powerful CEOs influence the board to over-invest in risky projects for high returns, which increases the firm’s risk and enhances the firm’s contribution to systemic risk. The results obtained are also similar to the findings of Altunbaş et al. (2020) that CEO power is positively correlated with risk-taking.

Table 3 shows CEO overconfidence and CEO power effect on systemic risk for non-bank financial institutions (NBFIs). Model (1) presents the results for overall NBFIs, indicating that CEO overconfidence has a positive but insignificant effect on systemic risk, whereas CEO power enhances the systemic risk with coefficient values of 0.019. Moreover, the results indicate that the control variables size and leverage are positively associated with systemic risk. Model (2) show the results for state-owned non-banking financial institutions, indicating that CEO power is significantly positively linked to systemic risk with a coefficient value of 0.028. In model (3) for non-state-owned non-banking financial institutions, the results are positive but insignificant for CEO power and systemic risk. The results demonstrate that for non-banking financial institutions, organizations with powerful CEOs contribute more to systemic risk, whereas CEO overconfidence results are insignificant. The logical reasoning for this could be that the Chinese government highly regulates non-bank financial institutions, and large enterprises are owned by the government. The positive effect of CEO power on systemic risk indicates that powerful CEOs adopt policies that lead to more risky results and guide board decisions to adopt risky policies (Adams et al., 2005). Powerful CEOs influence the board to over-invest in risky projects for high returns, which increases the firm’s risk and increases the firm’s contribution to systemic risk. These results are similar to the findings of Altunbaş et al. (2020). Table 3 results also demonstrate that non-bank financial institutions’ size is significantly positively linked with systemic risk, demonstrating that the bigger the firm size, the higher the systemic risk. These findings are also consistent with earlier studies (De Jonghe et al., 2015; Kleinow and Nell, 2015).

**Table 3:** Regression analysis for non-bank financial institutions (NBFIs).

	Dependent variable: systemic risk ( ΔCoVaR )		
	(1) Non-banking financial institutions	(2) SOE (NBFIs)	(3) Non-SOE (NBFIs)
OCE_t_	0.047	0.107	0.027
	(0.120)	(0.099)	(0.085)
PWE_t_	0.019^**^	0.028^**^	0.013
	(0.010)	(0.014)	(0.011)
Size_t_	0.307^**^	0.43^***^	0.177^***^
	(0.142)	(0.125)	(0.062)
LEVR_t_	0.092^**^	0.146^*^	0.030
	(0.041)	(0.081)	(0.045)
ROA_t_	−0.012^***^	−0.07	−0.142^**^
	(0.004)	(0.123)	(0.068)
LNR_t_	0.060	0.048	0.093
	(0.058)	(0.098)	(0.181)
N	1898	1,090	808
adj. R2	0.246	0.361	0.263
Hausman test (value of *p*)	0.001	<0.001	<0.001
F (*P*-value)	<0.001	0.004	0.006

The asterisks *, **, and *** indicate the significance levels at 10%, 5%, and 1%, respectively.

Table 4 gives the robustness test results of the two-stage least squares regression method. We adopted the instrument variable method to verify the results and control for any endogeneity concerns based on previous research studies (Shahab et al., 2020; Safi et al., 2021). We used the mean value of CEO overconfidence based on the province as an instrumental variable for the 2-SLS method. The results further confirm that CEO overconfidence enhances the systemic risk contribution in the context of banks, whereas CEO power positively affects a firm’s systemic risk contribution for non-bank financial institutions of China. These results are similar to the previously obtained results. Moreover, these results are supported by the studies of Lee et al. (2020) and Liu et al. (2020).

**Table 4:** Regression analysis (2-SLS method).

	First stage	Second stage	
	(1) OCE_t_	(2) Banks (ΔCoVaR)	(3) Non-bank financial institutions ( ΔCoVaR )
Instrument_OCE_t_	0.432^**^	0.294^*^	0.036
	(0.228)	(0.151)	(0.704)
PWE_t_	0.052	0.114	0.025^***^
	(0.309)	(0.410)	(0.001)
Size_t_	0.281^**^	0.179^***^	0.548^**^
	(0.128)	(0.051)	(0.229)
LNR_t_	0.308^**^	0.322^***^	0.103^***^
	(0.139)	(0.073)	(0.004)
Levr_t_	−0.125^**^	0.013	0.01
	(0.061)	(0.030)	(0.527)
ROA_t_	0.989^*^	−0.093	−0.087^***^
	(0.565)	(0.207)	(0.017)
N	2,708	810	1898
F	0.001	<0.001	<0.001

OCE shows CEO overconfidence, Instrument_OCE is the instrumental variable created using average provincial CEO overconfidence, PWE represents CEO Power, Size is the firm’s size, Levr is the leverage ratio, ROA is the returns on assets, and LNR is the loan ratio of the firm. The asterisks *, **, and *** indicate the significance levels at 10%, 5%, and 1%, respectively.

## Conclusion and recommendations

The financial crisis of 2007–2008 exposed the financial system’s fragility when a decline in the financial sector affected other sectors and hindered overall economic growth. Moreover, the crises spread to other countries, slowed their economic growth, and led governments, monitoring agencies, and researchers to focus on systemic risk and identify the key factors that add to systemic risk. Previous studies have ignored the behavioral perspective, including CEO power and overconfidence. Thus, the present study investigates the association between CEO overconfidence, CEO power, and systemic risk. We obtained data for banks and non-bank financial institutions operating in the Shenzhen and Shanghai Stock exchanges from 2006 to 2018. We measured financial institutions’ contributions toward systemic risk using the delta conditional value at risk method. The results indicate that the systemic risk contribution of non-banking financial institutions is higher compared to banks. The results of this study show that CEO overconfidence significantly increases the firm’s systemic risk contribution for banks, whereas CEO power is significant and positive only for non-state-owned banks’ systemic risk. For non-bank financial institutions, the association between CEO overconfidence and systemic risk was insignificant, whereas powerful CEOs in the non-banking financial sector significantly increased the firm’s systemic risk contribution. Furthermore, the results show that for both banks and non-bank financial institutions, firm size enhances its contribution to systemic risk. The results obtained were robust-check by the two-stage least squares method, which further confirms the previous results.

The present study has important policy implications for firms, policymakers, and regulators as we have identified systemically important banks and non-bank financial institutions. Moreover, the present study has identified important elements that influence systemic risk. However, this study’s findings do not mean that overconfident CEOs are detrimental or should be avoided, as previous studies conducted found firms with overconfident CEOs as beneficial and innovative (Kim et al., 2016). Though, regulators and policymakers can enforce mechanisms like conservative accounting methods to restrain CEOs from overestimating a project to minimize the effect of CEO overconfidence on systemic risk. To minimize the impact of CEO power on systemic risk, non-bank financial institutions should increase their independent board size and institutional investors. By including independent board directors, the firm will reduce the contribution of CEO power to systemic risk.

## Data availability statement

The data analyzed in this study is subject to the following licenses/restrictions: the datasets used for this study’s analysis can be obtained from the Wind Financial database (https://www.wind.com.cn/en/wft.html), and China Stock Market Accounting Research (CSMAR) can be accessed at www.gtadata.com. The CSMAR and WIND data in this study is used under a license and is subject to restrictions. Requests to access these datasets should be directed to https://www.wind.com.cn/en/wft.html and www.gtadata.com.

## Author contributions

AS and YC presented the current paper’s idea with the co-author’s help, designed the methodology section, and simulated and listed the results. YZ appreciated the idea and helped draft and, particularly, revise the manuscript, where they put forward several modifications and amendments at different stages. All authors contributed to the article and approved the submitted version.

## Conflict of interest

The authors declare that the research was conducted in the absence of any commercial or financial relationships that could be construed as a potential conflict of interest.

## Publisher’s note

All claims expressed in this article are solely those of the authors and do not necessarily represent those of their affiliated organizations, or those of the publisher, the editors and the reviewers. Any product that may be evaluated in this article, or claim that may be made by its manufacturer, is not guaranteed or endorsed by the publisher.
